# Air entrapment as a potential cause of early subcutaneous implantable cardioverter defibrillator malfunction: a systematic review of the literature^[Author-notes euac046-FM1]^

**DOI:** 10.1093/europace/euac046

**Published:** 2022-03-25

**Authors:** Hussam Ali, Pierpaolo Lupo, Sara Foresti, Guido De Ambroggi, Carmine De Lucia, Diego Penela, Dario Turturiello, Edoardo Maria Paganini, Riccardo Cappato

**Affiliations:** Arrhythmia & Electrophysiology Center, IRCCS—MultiMedica Group, Via Milanese 300, 20099 Sesto San Giovanni, Milan, Italy; Arrhythmia & Electrophysiology Center, IRCCS—MultiMedica Group, Via Milanese 300, 20099 Sesto San Giovanni, Milan, Italy; Arrhythmia & Electrophysiology Center, IRCCS—MultiMedica Group, Via Milanese 300, 20099 Sesto San Giovanni, Milan, Italy; Arrhythmia & Electrophysiology Center, IRCCS—MultiMedica Group, Via Milanese 300, 20099 Sesto San Giovanni, Milan, Italy; Arrhythmia & Electrophysiology Center, IRCCS—MultiMedica Group, Via Milanese 300, 20099 Sesto San Giovanni, Milan, Italy; Arrhythmia & Electrophysiology Center, IRCCS—MultiMedica Group, Via Milanese 300, 20099 Sesto San Giovanni, Milan, Italy; Arrhythmia & Electrophysiology Center, IRCCS—MultiMedica Group, Via Milanese 300, 20099 Sesto San Giovanni, Milan, Italy; Arrhythmia & Electrophysiology Center, IRCCS—MultiMedica Group, Via Milanese 300, 20099 Sesto San Giovanni, Milan, Italy; Arrhythmia & Electrophysiology Center, IRCCS—MultiMedica Group, Via Milanese 300, 20099 Sesto San Giovanni, Milan, Italy

**Keywords:** S-ICD, Inappropriate shock, Air entrapment, Oversensing, Systematic review

## Abstract

**Aims:**

Air entrapment (AE) has been reported as a potential cause of early inappropriate shocks (ISs) following subcutaneous implantable cardioverter defibrillator (S-ICD) implantation, but a cause–effect relationship is not always evident. This systematic review aims to analyse this phenomenon concerning implantation techniques, electrogram (EGM) features, radiologic findings, and patient management.

**Methods and results:**

A systematic search was conducted using PubMed, Embase, and Google Scholar databases following the PRISMA guidelines to obtain all available literature data since 2010 on S-ICD malfunctions possibly due to AE. The final analysis included 54 patients with AE as a potential cause of S-ICD malfunction. Overall, the aggregate incidence of this condition was 1.2%. Of ICD malfunctions possibly due to AE, 93% were ISs, and 95% were recorded within the first week following implantation. Radiologic diagnosis of AE was confirmed in 28% of the entire study cohort and in 68% of patients in whom this diagnostic examination was reported. At the time of device malfunction, EGMs showed artefacts, baseline drift, and QRS voltage reduction in 95, 76, and 67% of episodes, respectively. Management included ICD reprogramming or testing, no action (observation), and invasive implant revision in 57, 33, and 10% of patients, respectively. No recurrences occurred during follow-up, irrespective of management performed.

**Conclusions:**

Device malfunction possibly due to AE may occur in ∼1% of S-ICD recipients. Diagnosis is strongly suggested by early occurrence, characteristic EGM features, and radiologic findings. Non-invasive management, principally device reprogramming, appears to be effective in most patients.

**What’s new?**

- Device malfunctions possibly due to air entrapment may occur in ∼ 1% of subcutaneous implantable cardioverter defibrillator patients.
- They are mostly inappropriate shocks in the early days after implantation, but defibrillation abnormalities may be observed.
- The diagnosis is mainly based on early occurrence, typical electrogram features, and radiological findings.
- Non-invasive management, principally device reprogramming or observation, is effective in most patients.
- Further studies are required to establish this phenomenon’s actual incidence and clinical impact.

## Introduction

The subcutaneous implantable cardioverter defibrillator (S-ICD) has been introduced in clinical practice to prevent sudden cardiac death with approved safety and efficacy profiles.^1^ Similarly with transvenous ICDs, inappropriate shocks (ISs) represent a major concern regarding S-ICD therapy, negatively impacting morbidity and mortality.^2,3^

While the leading cause of ISs with the transvenous ICD is supraventricular arrhythmias (e.g. atrial fibrillation), they are mostly related to myopotentials or T-wave oversensing in S-ICD patients.^3,4^ Following the expansion of S-ICD in clinical practice, uncommon causes of ISs, such as air entrapment (AE) in the subcutaneous space, have been observed. Since its first description by Zipse *et al*.^5^ in 2014, various S-ICD malfunctions possibly due to AE have been reported early after implantation. Nonetheless, a direct cause–effect relationship between AE and S-ICD malfunction is not always evident. Moreover, a systematic collective review of this phenomenon is lacking. This study aims to collect and analyse the available literature data concerning clinical details, implantation techniques, electrogram (EGM) features, radiologic findings, and management of S-ICD malfunctions possibly due to AE.

## Methods

The study was performed in accordance with guidelines from the *Preferred Reporting Items for Systematic Reviews and Meta-Analyses* (PRISMA).^6^ Considering the elusive nature of the analysed condition and the difficulty to prove a cause–effect relationship between AE and many of the reported S-ICD malfunctions, we referred to these cases throughout the entire text as *possibly due to AE*. PubMed, Embase, and Google Scholar databases were searched for articles published in the period from 1 January 2010 to 31 August 2021 containing the combination of ‘air’ *and* ‘subcutaneous’ in the Full Text *and any* of the following terms in the Title: S-ICD, ICD, defibrillator, cardioverter, or ‘inappropriate shock’. Subsequently, any published data, including clinical studies, registries, case reports, abstracts, or short communications, were considered eligible for the study analysis when satisfying all of the following criteria: (i) reported S-ICD malfunction or IS possibly due to AE or air noise; (ii) published in a peer-reviewed journal; and (iii) available in the English language. Further analysis of the biography of qualified articles was also performed to include additional potentially eligible cases. Every effort was made to avoid duplicated or overlapping data, focusing on the authors/centre, patient clinical data, and EGM recordings when available. Finally, two unpublished cases (authors’ data) of S-ICD malfunctions possibly due to AE were also included. Literature screening, data selection/extraction, and EGM analysis were validated by two cardiac electrophysiologists (H.A. and P.L.). Whenever occurred, disagreement was resolved by a consensus or a third judgement (R.C.).

Case reports were analysed with a focus on the following data, when available: clinical setting, S-ICD implantation technique and programming, type and timing of S-ICD malfunction, the assumed localization of AE, EGM features, chest X-ray (CXR) findings, clinical management, and follow-up. Conversely, studies including a cohort of S-ICD patients with less detailed information were mainly analysed to assess this phenomenon’s incidence when at least a short-term follow-up (≥3 months) was available.

Regarding EGM analysis, the following definitions were applied:

1. Artefacts: ≥2 abrupt, sharp (spike-like), or rounded deflections from the baseline with subsequent oversensing (non-cardiac signals).
2. Baseline drift: gradual deviation (wandering) of the isoelectric baseline for ≥1 large box amplitude (at the recording gain) and lasting ≥1 s.
3. Reduced QRS voltage: mute (standstill) line, hardly visible intrinsic QRS complexes at the recording gain, and/or ≥30% reduction of QRS voltage (compared with peri-shock EGMs, when available).

Finally, the potential *corrective* effect of shock on EGM abnormalities was also analysed.

## Results

**Figure 1:**
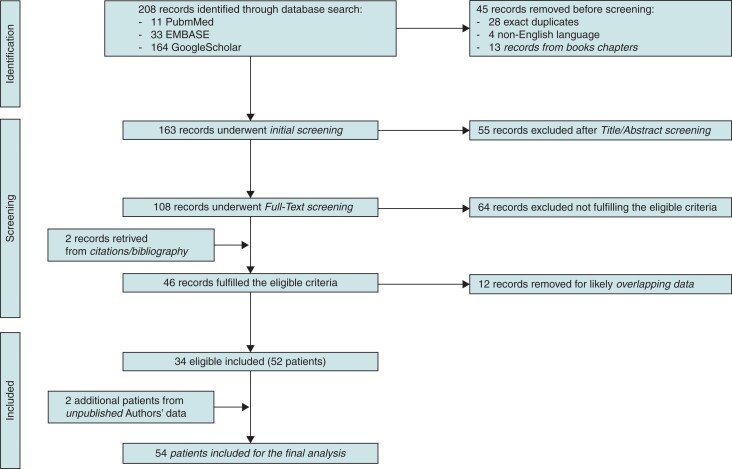
PRISMA flow diagram of database search. PRISMA flow diagram of database search and data extraction of the current review.

Initially, 208 records were identified through database search, of which 34 were eligible for the current analysis^5,7–39^ reporting 52 patients. After adding two unpublished cases from the authors’ experience, a total of 54 cases of S-ICD malfunction possibly due to AE were included. *Figure 1* shows a PRISMA flow diagram of database search, whereas Supplementary material online, *Table S1* summarizes the clinical and technical data of the analysis cohort.

### Incidence

Fourteen studies,^9,11,5–7,20,21,26,1–5,39^ with at least a short-term follow-up (≥3 months), reported the incidence of this phenomenon in different S-ICD cohorts, ranging from 0.4% in the Austrian registry^15^ to 8.3% in a small Polish cohort.^20^ Aggregating data from these 14 studies, 31 of 2433 (1.2%) S-ICD recipients experienced device malfunction possibly due to AE during the reported follow-up.

### Implantation technique, defibrillation threshold test, and device programming

Implantation technique, described in 18 patients, was performed using three and two incisions in 6 (33.3%) and 12 patients (66.6%), respectively. Defibrillation threshold test (DFT), reported in 19 patients, was unremarkable in 15 patients (78.9%). There were DFT-related issues in the remaining four patients (21%), such as ventricular fibrillation (VF) detection delay or inhibition, and unsuccessful DFT requiring pocket revision for suspected AE.^16,24,25^ In another patient, DFT had to be performed 1 week after S-ICD generator replacement due to unsatisfactory high impedance of the 10J shock test during the procedure.^37^ Sensing mode, specified in 22 patients, was programmed to the primary, secondary, and alternate vector in 7 (31.8%), 11 (50%), and 4 patients (18.1%), respectively. A conditional shock zone was activated in all 11 patients (100%) in whom the detection cut-off was mentioned.

### Type and timing of air entrapment-related subcutaneous implantable cardioverter defibrillator malfunction

**Figure 2:**
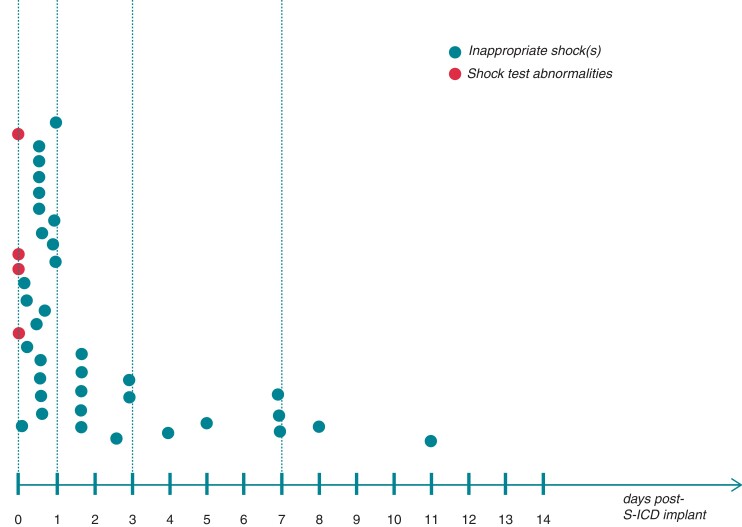
Type and timing of S-ICD malfunctions. A diagram showing the type and temporal distribution of S-ICD malfunctions possibly due to air entrapment. AE, air entrapment.

Of 54 patients, 50 (92.5%) had at least one IS, whereas four (7.4%) patients had DFT-related issues, manifested as inadequate VF detection in two,^24,25^ unsuccessful DFT in one,^16^ and high impedance of the shock test at device replacement in one (*Figure 2*).^37^ Event timing was available in 39 patients (72.2%). The undesired event occurred within 24, 72 h, and 1 week following S-ICD implantation in 24 (61.5%), 32 (82%), and 37 patients (94.8%), respectively (*Figure 2*). No AE-related ISs were observed beyond the 11th day following the procedure.^38^

### Radiologic findings and air entrapment localization

Of 22 patients in whom radiographic (CXR) or fluoroscopic data were available, AE was documented in 15 patients (68.1%), accounting for 27.7% of the entire study cohort (see Supplementary material online, *Figure S1*). Radiologic localization of AE, reported in 14 patients, was at the level of the distal electrode, proximal electrode, and device pocket in two (14.2%), seven (50%), and five patients (35.7%), respectively. Radiologic follow-up in patients with early AE was available in nine patients showing AE absorption within 2 weeks in eight patients (88.8%). In the remaining patients, complete AE resolution was documented at 1-month CXR control.^5^ Lateral CXR detected AE around the lead electrodes in eight patients, whereas anteroposterior CXR documented AE around the device in five patients.

Localization of AE was confirmed (radiologically) or presumed by analysing EGM features in different sensing vectors in 33 patients. The air entrapment site was adjacent to the proximal electrode, distal electrode, device pocket, and lead connector (set screw) in 10 (30.3%), 7 (21.2%), 7 (21.2%), and 9 patients (27.2%), respectively.

### Electrogram features at the time of malfunction

The EGM showing S-ICD malfunction was available in 21 patients (38.8%) (see Supplementary material online, *Figure S1*). Artefacts were present in 20 patients (95.2%). In all four patients with presumed AE in the set screw who had available EGMs, artefacts were monomorphic and repetitive, simulating ventricular tachyarrhythmias.^13^ Baseline drift and reduced QRS voltage were present in 16 (76.1%) and 14 patients (66.6%), respectively. Provocative manoeuvres described in 14 patients could replicate the EGM abnormalities at the time of device interrogation only in six patients (42.8%). Following shock delivery, the EGM was nearly normalized in nine patients (42.8%).

### Clinical management and follow-up

Clinical management of early S-ICD malfunction was described in 30 patients (55.5%) (see Supplementary material online, *Figure S2*). Due to delayed evaluation of a self-limiting phenomenon or inability of ICD reprogramming, no specific action was adopted in 10 patients (33.3%). Implantable cardioverter defibrillator reprogramming or testing was adopted in 17 patients (56.6%), including reprogramming the sensing vector to exclude the involved AE site, temporary ICD deactivation, and repeated DFT in 13 (43.3%), 5 (16.6%), and 2 patients (6.6%), respectively. After radiologic resolution of AE around the generator, DFT was repeated in one patient 2 days later following an IS associated with high shock impedance (151 ohms).^28^ In another patient, DFT was successfully performed 1 week after S-ICD generator replacement due to unsatisfactory high impedance of the 10J shock test at the time of procedure.^37^ Invasive implant revision was performed in three patients (10%), including lead repositioning, pocket revision, and unnecessary pocket inspection for suspected lead connection problems in the set screw. Follow-up data of these patients, with variable or undetermined durations, were reported in 22 patients (73.3%), with at least short-term follow-up (≥3 months) available in 10 patients (33.3%). Irrespective of the adopted management, no recurrences of S-ICD malfunction occurred during the reported follow-up.

## Discussion

While uncommon, malfunction of implantable cardiac devices due to AE is not a novel observation. About four decades ago, unipolar pacemaker malfunction as sensing failure or loss of capture was reported to be secondary to AE.^40,41^ Air entrapment in the set screw (lead connector) of the transvenous ICD may also lead to inappropriate sensing.^42^ Likewise, in the early days after implantable loop recorder placement, intermittent loss of device-tissue contact and AE may cause similar EGM abnormalities with inappropriate sensing and false asystole.^43^ Notably, the anatomical nature of the S-ICD system, with sensing electrodes located in the subcutaneous space or within surgical pockets, makes the sensing process prone to undesired interference with AE during the acute phase after implantation. Although uncommon and self-limiting, early ISs related to AE occurring just within a few hours after the procedure or hospital discharge may have a negative impact on acceptance of device therapy and future compliance of affected patients.

### Incidence

The actual incidence of S-ICD malfunction due to AE is unknown and accurate estimation is challenging. In this review, analysing only studies reporting S-ICD malfunction possibly due to AE, overestimation of its incidence cannot be excluded. In our analysis, aggregate data from different S-ICD cohorts revealed an incidence of 1.2% although several large S-ICD studies and registries^4–6^ were not included since they did not refer directly to AE as a potential cause of S-ICD malfunction. However, AE might play a role in the genesis of *non-cardiac oversensing* associated with ISs that have been reported with a variable incidence: 1.6% in the S-ICD post-approval study,^44^ 1.4% in the UNTOUCHED trial,^45^ and 2.2% in the EFFORTLESS registry.^46^

Moreover, a considerable proportion of ISs in the early phase following S-ICD implantation may be related to AE. In the Maude registry, AE in S-ICD patients was the cause of 8% of reported oversensing episodes.^47^ Likewise, in a systematic review of the literature, Santomauro *et al*.^48^ analysed the aetiology of extracardiac ISs in 2654 S-ICD patients in whom AE accounted for 23% of ISs related to oversensing of non-cardiac signals.

### Implantation technique, defibrillation threshold test, and device programming

Reported S-ICD malfunctions occurred with both two- and three-incision techniques. However, none of the patients implanted with the three-incision technique had confirmed AE around the distal electrodes, whereas AE at this site was confirmed radiologically in two patients who underwent S-ICD implantation using the three-incision technique. Accordingly, the two-incision technique might reduce the risk of AE around the distal electrode by eliminating the superior parasternal surgical pocket. However, some authors suggest that this technique may expose the lead tip to an increased dislocation risk and possible formation of adjacent AE.^10^

Notably, the incidence of S-ICD malfunction possibly due to AE was highly variable between studies irrespective of their time frame. This is likely due to differences in sample size, patients’ characteristics, surgical technique, and the centre/operator experience. However, some author groups^17,33^ reported avoiding this complication in subsequent patients after optimizing their surgical implantation technique. For instance, saline flushing and skin massage of the xiphoid and device pockets before surgical closure should reduce the formation of AE.

Although activation of a conditional shock zone has become a common practice to reduce ISs in S-ICD patients,^46^ it did not seem to reduce ISs related to AE in our study. This is not surprising, as EGM components during AE oversensing do not match the sinus rhythm template, and the sensed events intervals often occur within the VF zone where there is no role for the discriminative algorithms. Likewise, DFT was unremarkable in most patients, highlighting its poor predictive value regarding subsequent AE-related issues. Finally, and due to the features of related EGM abnormalities, the SMART Pass filter, mainly developed to reduce T-wave oversensing, is not expected to reduce IS possibly related to AE.

### Type and timing of subcutaneous implantable cardioverter defibrillator malfunctions

Most malfunctions were ISs, with >90% of patients experiencing this complication. However, only a few patients suffered multiple (>2) ISs or inappropriate ICD storms,^5,30,34,36^ which is not uncommon in patients with ISs due to lead fracture or failure. The transient nature of AE and the potential corrective effect of shock delivery on related EGM abnormalities might explain this finding.

Moreover, in a minority of patients (7.4%), DFT issues related to AE were described. Air entrapment should be suspected during DFT when there is inadequate VF detection or high shock impedance despite optimal S-ICD positioning. Careful EGM analysis and fluoroscopic check may reveal AE as a potential cause and guide management.

The early occurrence represents the most characteristic feature of S-ICD malfunctions possibly due to AE. In the current analysis, it occurred within the first week following S-ICD implantation in most patients (∼95%) with no ISs reported beyond the 11th day after implantation. Therefore, AE should be considered as a potential cause of ISs during the early days after implantation, whereas it is unlikely to be the underlying mechanism when occurring beyond the first week. This is consistent with the physiologic absorption and resolution of residual air in the subcutaneous space. Interestingly, in a small study evaluating the amount and absorption rate of AE in S-ICD patients using CT imaging, the mean AE immediately after implantation was ∼28 mL with an absorption rate of 99% at 1 week after the procedure.^49^ Of note, in the patient with reported ISs 11 days after implantation, the readmission CXR showed 90° rotation of pulse generator, and thus other mechanisms cannot be excluded.^38^

### Radiologic findings and the cause–effect relationship between air entrapment and device malfunction

Radiologic confirmation of AE was reported only in 27.7% of patients in our cohort and could not be detected in >30% of patients in whom radiologic inspection was reported. This might be due in part to the transient and dynamic nature of AE in the subcutaneous space, but it raises the question about the actual cause–effect relationship between AE and early S-ICD malfunction. Air entrapment after S-ICD implantation is probably more frequent than expected and might represent a bystander condition at the time of early ISs or abnormal DFT. Of note, AE in the set screw (lead connector) cannot be detected radiologically, and the diagnosis was based on the early occurrence, EGM analysis, and the subsequent reversal of the condition during the following days, consistent with AE absorption. Although the mechanism of these cases remains unclear, incorrect insertion of the wrench tool or the lead tip may play a role, as has been reported with transvenous ICD.^42^ Interestingly, all available EGMs of presumed AE in the set screw showed repetitive monomorphic artefacts with subsequent EGM normalization following shock delivery, simulating appropriate therapy. However, mechanical damage to the seal plug of the pulse generator might produce similar effects.^50^ Other mechanisms, such as muscular contractions, movement artefacts in the early days after the procedure caused by unstable electrodes-tissue contact, cannot be fully excluded. In patients with previous cardiac surgery, electrical interference with sternal wires might cause ISs.^20^ Morani *et al*.^51^ described an S-ICD patient who suffered from IS due to an unusual tunnelling course beneath the sixth rib, and intermittent mechanical stress on the lead was hypothesized to be the cause of repetitive monomorphic artefacts analogous to those observed in patients with AE in the set screw.

Air entrapment resolved in all patients with confirmed AE and available radiologic follow-up, showing an absorption rate of about 90% within 2 weeks, which corroborates the early and transient nature of this phenomenon.^49^ Notably, lateral CXR was most helpful in detecting AE around the lead electrodes, whereas the anteroposterior projection helped to detect AE around the device, highlighting the importance of obtaining orthogonal CXRs after implantation.

### Electrogram features

**Figure 3:**
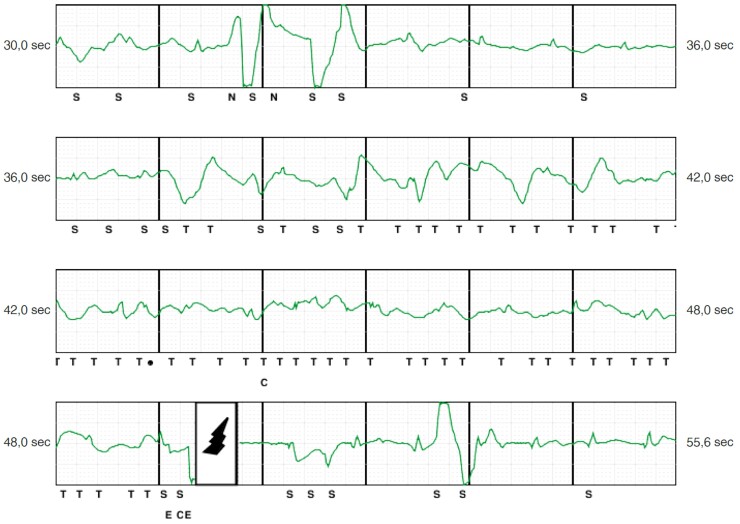
Inappropriate S-ICD shock due to air entrapment around sensing electrodes. Representative EGM of an inappropriate shock occurring 24 h after S-ICD implantation due to air entrapment around the proximal electrode. Typical EGM features include artefacts, baseline drift, and QRS voltage reduction.

**Figure 4:**
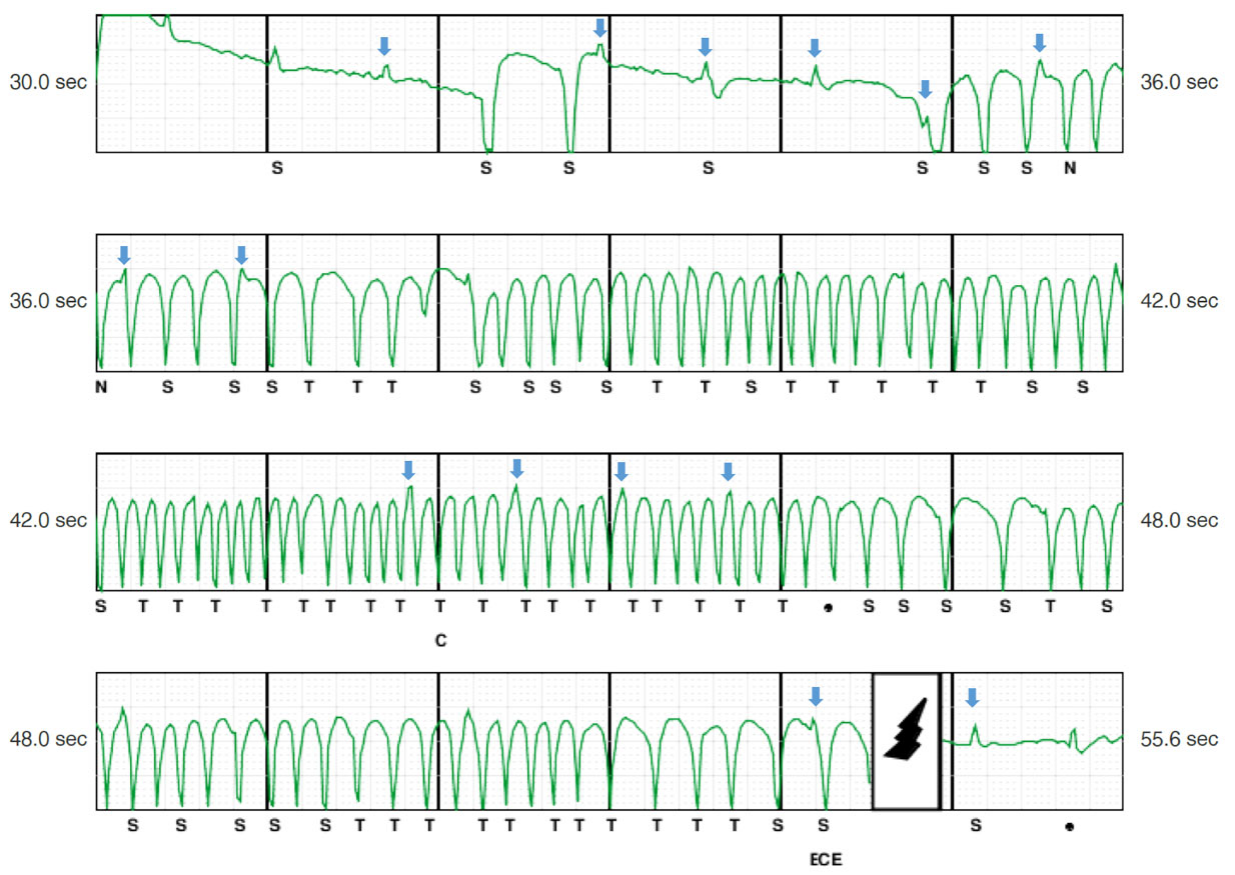
Inappropriate S-ICD shock due to air entrapment in the set screw. Representative EGM of an inappropriate shock occurring 72 h after S-ICD implantation likely related to air entrapment in the set screw. After initial baseline drift, typical EGM features show repetitive monomorphic artefacts and post-shock EGM normalization simulating an appropriate therapy. Arrows indicate intrinsic QRS complexes.

Despite provocative manoeuvres, EGM abnormalities were reproducible only in 40% of patients at the time of device interrogation, adding further diagnostic difficulties and reflecting the potential intermittent and transient effects of AE on sensing process. Following shock delivery, the EGM nearly normalized in about 40% of cases, likely due to AE redistribution following shock, which may reduce the risk of subsequent inappropriate storms.^28^ Oversensing related to AE is mainly due to artefacts sensing and/or auto-gain function following QRS voltage reduction. During episodes, the most common findings were artefacts (95.2%), followed by baseline drift and reduced QRS voltage in 76.1 and 66.6%, respectively. Analysing EGMs of patients with presumed AE in the lead connector showed repetitive monomorphic artefacts in all cases with subsequent EGM normalization following S-ICD shock, simulating an appropriate therapy for ventricular tachyarrhythmia.^13^*Figures 3* and *4* show representative EGMs of ISs related to AE around the electrodes and the set screw, respectively.

### Management and preventive measurements

Reprogramming the sensing vector to exclude the potential AE was effective in more than 40% of patients. This programming flexibility is meaningful to overcome sensing issues compared with conventional ICDs. Moreover, in one-third of patients, no specific action was performed, and a ‘wait and see’ strategy was adopted probably due to delay in evaluation or inability of ICD reprogramming. Implantable cardioverter defibrillator therapy was temporarily deactivated in 16.6% of patients to avoid further ISs. Notably, 10% of patients underwent invasive implant revision (pocket revision, lead repositioning) due to AE-related malfunction. When AE is suspected, it is advisable to avoid invasive management since this condition is typically self-limiting and reprogramming the sensing vector, temporal ICD deactivation, and/or a ‘wait and see’ strategy are usually efficient measures. Remarkably, no recurrences of S-ICD malfunction occurred during the reported follow-up, irrespective of the adopted management.

**Figure 5:**
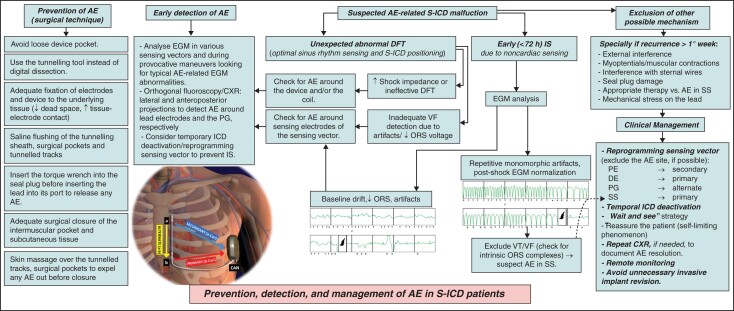
Prevention and management of S-ICD malfunctions possibly due to air entrapment. A proposed approach for prevention, detection, differential diagnosis, and management of AE and possible related malfunctions in S-ICD patients. AE, air entrapment; CXR, chest X-ray; DE, distal electrode; DFT, defibrillation threshold test; EGM, electrogram(s); IS, inappropriate shock(s); PE, proximal electrode; PG, pulse generator; S-ICD, subcutaneous ICD; SS, set screw; VF, ventricular fibrillation; VT, ventricular tachycardia.

Many authors,^12^ and Boston Scientific manufacturer, recommend optimal surgical techniques to reduce the risk of AE during S-ICD implantation. Adequate electrodes fixation to minimize the dead space and improve electrode-tissue contact, saline flushing of the pockets and sternal track, avoiding blunt digital dissection, and gentle skin massage over the tunnelled track, the xiphoid and device pockets to expel any residual air out before surgical closure are advisable manoeuvres and may minimize the risk of AE formation during the procedure. Moreover, to avoid AE in the header or mechanical damage to the seal plug, it is recommended both leaving the torque wrench in place when inserting the lead, ensuring that the electrode tip is fully inserted in the header, and also inserting the torque wrench at a 90° angle into the set screw with the S-ICD generator held flat. After implantation, careful device interrogation, analysing sensing vectors during provocative manoeuvres, and reviewing orthogonal CXRs help to recognize and localize potential AE and guide judicious management. Finally, immediately activated after implantation, the remote monitoring may allow for early detection of AE-related abnormalities. *Figure 5* provides a proposed approach for prevention, early detection, differential diagnosis, and management of AE in S-ICD patients.

### Study limitations

First, and due to the retrospective nature of data collection, clinical and technical details were incomplete in many patients. Cohort studies, for instance, with no detailed information, were mainly included to assess the approximate incidence of this condition. Secondly, analysing only studies reporting AE as a potential cause of S-ICD malfunction, overestimation of its incidence cannot be excluded; however, the included cohort studies represent various S-ICD populations, with a considerable aggregate number of patients treated in different centres. Thirdly, the accuracy of EGM analysis might be suboptimal due to the lack of complete EGM recordings and the use of arbitrary criteria to define EGM abnormalities. Fourthly, no specific statistical analysis could be performed considering the non-solid nature of data derived from case reports, and thus the results were simply expressed in percentile (%) when applicable. Accordingly, this review aims to highlight some clinical and technical aspects of this condition rather than to derive definitive conclusions.

## Conclusions

Whilst a cause–effect relationship is not always evident, AE may be associated with early S-ICD malfunction, mostly ISs occurring within the first days after implantation. Diagnosis is mainly based on early occurrence, characteristic EGM features, and radiologic findings. Non-invasive management, including device reprogramming or observation, appears to be effective in most patients. Prompt recognition may help to avoid further ISs and unnecessary invasive implant revisions. Further studies are required to establish the actual incidence and clinical impact of this phenomenon.

## Supplementary material

Supplementary material is available at *Europace* online.

## Supplementary Material

euac046_Supplementary_Data

## Data Availability

The data underlying this article will be shared on a reasonable request to the corresponding author.
