# Supplementary material for: Air entrapment as a potential cause of early subcutaneous implantable cardioverter defibrillator malfunction: a systematic review of the literature
Source: Europace. 2022 Mar 25;24(10):1608–16. doi: 10.1093/europace/euac046 (PMC9757921; doi:10.1093/europace/euac046)
Supplement: euac046_Supplementary_Data [file euac046_supplementary_data.docx]

**Table S1:** **Clinical and technical available data of study patients**

| **Pt** | **Reference** | **Clinical setting** | **Implant technique, DFT, programming** | **Malfunction type / timing** | **Sensing vector:**  **EGM features** | **Possible AE site** | **Radiologic findings** | **Clinical management** | **Follow-up** |
| --- | --- | --- | --- | --- | --- | --- | --- | --- | --- |
| 1 | 2014 [5] | 56 M, IHD,  II° prevention | 3-incision, DFT OK | 7 ISs /< 72 h | II° vector: baseline drift, ↓QRS, artifacts; reproducible | DE | - AE around DE (lateral CXR); resolved ≤ 1 mos | - temporary ICD deactivation  - reprogramming to I° vector | negative FU (NDD) |
| 2 | 2014 [7] | 22 F, idiopathic PMVT, II° prevention | 2-incision | 2 ISs / 2 h | I° vector: EGM NA.  reported ↓QRS, artifacts | PE | - AE around PE | unnecessary pocket revision (suspected lead connection issue) | NA |
| 3 | 2015 [8] | 37 M, Brugada,  II° prevention | 3-incision, DFT OK | 2 ISs /< 24 h | alt vector: baseline drift, ↓QRS, artifacts; reproducible | PE | - AE around PE (lateral CXR); resolved ≤ 10 d | no action | negative mid-term FU |
| 4-5 | 2016 [9] | 2/86 Pt | NA | IS | NA | NA | NA | NA | NA |
| 6 | 2016 [10] | 56 M, IHD | 2-incision, NO DFT, CSZ 190-230 bpm | 1 IS / < 24 h | alt vector: baseline drift, ↓QRS, artifacts; not reproducible | DE or PE | No AE | no action | negative FU (NDD) |
| 7 |  | 73 M, valvular HD,  II° prevention | 2-incision, NO DFT  CSZ 200-250 bpm | 1 IS / < 24 h | II° vector: baseline drift, ↓QRS, artifacts; not reproducible | DE | No AE, anterior displacement of DE | reprogramming to I° vector |  |
| 8 | 2016 [11] | 35 M,TGA (Mustard) (1/21 GUCH Pt) | NA | 1 IS / < 24 h | baseline drift, ↓QRS, artifacts, post-shock EGM normalization | NA | NA | NA | NA |
| 9 | 2017 [12] | 55 M, HCM, NSVT, I° prevention | 3-incision, DFT OK | IS / 6 h | I° vector: baseline drift, ↓QRS, artifacts; post-shock EGM normalization; not reproducible | PE | AE around PE (lateral CXR); resolved ≤ 2 wk | reprogramming to II° vector | negative 2 wk FU |
| 10 | 2017 [13] | 45 M, IHD, syncope  II° prevention | CSZ 180-220 bpm | 1 IS / 1 wk | alt vector: repetitive monomorphic artifacts; post-shock EGM normalization; not reproducible | SS | NA | no action (delayed evaluation) | negative 3 mos FU |
| 11 | 2017 [14] | 56 M, non-IHD,  I° prevention | 2-incision, DFT OK | 1 IS / < 48h | II° vector: baseline drift, artifacts; post-shock EGM normalization; reproducible | PG | AE beneath PG 1 d after implantation (AP-CXR), resolved 1 d after discharge | temporary ICD deactivation | negative FU (NDD) |
| 12 | 2017 [15] | 1/236 Pt  (Austria Registry) | NA | IS | EGM NA, reported artifacts | NA | NA | lead repositioning | NA |
| 13 | 2017 [16] | 1/36 Pt (2-incision, intermuscular) | 2-incision, unsuccessful DFT | unsuccessful DFT / during procedure | NA | PG | NA | pocket revision | negative 10 mos FU |
| 14-16 | 2017 [17] | 3/56 Pt | NA | IS / 1 d, 8 d, < 1 wk | NA | DE 2 Pt  SS 1 Pt | NA | NA | negative 11 mos FU |
| 17 | 2018 [18] | 17 F, LQTS  II° prevention | 3-incision; DFT OK | 1 IS /4 d | II° vector: baseline drift, artifacts, QRS ↔ | DE | AE around DE (lateral CXR); resolution 12 d | NA | NA |
| 18 | 2018 [19] | 75 M, Idiopathic  DCM, I° prevention | NA  CSZ 200-220 bpm | 1 IS /12 h | I° vector: baseline drift, ↓QRS, artifacts; reproducible | PE | no AE | temporary ICD deactivation  reprogramming to II° vector | negative 6 wk FU |
| 19 | 2018 [20] | 1/12 Pt, 70 M  IHD, previous cardiac surgery,  II° prevention | DFT OK  CSZ 200-220 bpm | 1 IS / < 24 h | II° vector: baseline drift, QRS ↔, repetitive monomorphic artifacts; post-shock EGM normalization | SS | no AE | reprogramming to I° vector | negative 13 mos FU |
| 20 | 2018 [21] | 1/75 Pt | NA | IS | NA | NA | NA | NA | NA |
| 21 | 2019 [22] | 58 F, chemotherapy induced DCM,  I° prevention | 2-incision; DFT OK CSZ 200-220 bpm | 1 IS / few h | I° vector: baseline drift, ↓QRS, artifacts; reproducible;  post-shock EGM normalization | PE | substernal AE (lateral CXR) | reprogramming to II° vector | negative 2 wk FU |
| 22 | 2019 [23] | 57 M, Brugada, syncope | 3-incision, DFT OK | 1 IS / 2 h | I° vector: baseline drift, ↓QRS, artifacts | PE | AE around PE (lateral CXR); resolved on 7 d | reprogramming to II° vector | NA |
| 23 | 2019 [24] | 40 M, DCM  II° prevention | 2-incision, abnormal DFT | during DFT/ delayed VF detection | alt vector: artifacts during induced VF and post-shock; not reproducible (next day) | PE | NA | temporary reprogramming to II° vector | NA |
| 24 | 2019 [25] | NA 1/570 Pt;  no follow-up | abnormal DFT | during DFT/ shock inhibition | EGM NA; VF undersensing 🡪 shock inhibition | DE or PE | AE around electrodes | NA | NA |
| 25-26 | 2019 [26] | 2/90 Pt (previous transvenous ICD extraction) | NA | IS | NA | DE | NA | no action | NA |
| 27 | 2019 [27] | 60 F, alcohol abuse,  hemodialysis,  II° prevention | DFT OK | 1 IS / 72 h | II° vector: repetitive artifacts | PG | AE beneath PG; resolved on 6 d (AP-CXR) | temporary ICD deactivation | negative FU (NDD) |
| 28 | 2019 [28] | 41 M, Brugada,  II° prevention | 3-incision, DFT effective (shock imp 98 ohm); CSZ 210-240 bpm | 1 IS / 24 h  ↑ shock imp | I° vector: ↓QRS, artifacts; post-shock EGM normalization; not reproducible | PG | AE around PG (AP-CXR), reduced on 2 d | repeated DFT after 2 d | negative FU (NDD) |
| 29 | 2020 [29] | 50 M, LV diverticula,  II° prevention | DFT OK | 1 IS / 1 wk | II° vector: repetitive monomorphic artifacts; post-shock EGM normalization | SS | No AE | reprogramming to I° vector | NA |
| 30 | 2020 [30] | 72 F, heart failure,  II° prevention | NA | multiple ISs / < 24 h | EGM NA:  reported ↓QRS and noise | PE | AE around PE (lateral CXR); resolved < 48 h | reprogramming to II° vector, pocket massage | negative FU (NDD) |
| 31 | 2020 [31] | 1/144 Pt (previous transvenous-ICD extraction) | NA | IS / 1 d | EGM NA:  reported artifacts | SS | NA | NA | NA |
| 32 | 2020 [32] | 1/98 Pt (19 Pt,  prior sternotomy) | NA | IS | NA | NA | NA | NA | NA |
| 33-37 | 2020 [33] | 5/238 Pts  1 pt with inherited arrhythmia syndrome | NA | IS / < 24h | EGM available in 1 case:  baseline drift, ↓QRS | PE and/or PG | NA | prevention next cases optimizing surgical technique | NA |
|  |  |  |  |  | NA | PG |  | no action |  |
| 38 | 2020 [34] | 1/48 Pt | 2-incision, DFT OK | multiple ISs / 5 d | EGM NA | PE | NA | reprogramming sensing vector | negative 3 mos FU |
| 39  40 | 2021 [35] | 2/39 Brugada Pt | CSZ ON | 1 IS | II° vector. EGM NA | SS | NA | no action | negative 26 mos FU |
| 41 | 2021 [36] | 29 M  sarcoidosis,  II° prevention | 2-incision, DFT OK | 5 ISs / < 48h | II° vector: baseline drift, ↓QRS, artifacts; reproducible | PG | AE around PG (AP CXR) | reprogramming to alt vector | negative FU (NDD) |
| 42 | 2022 [37] | 68 F, IHD | S-ICD generator replacement | ↑ imp of shock test (135 ohm at 10 J) | NA | PG | AE around PG (AP CXR) | ICD deactivation, delayed DFT to 7d (successful after imp drop) | negative 5 mos FU |
| 43 | 2021 [38] | 18 M, GUCH  II° prevention | 2-iniciosn  CSZ 200-220 bpm | 2 IS/ 11d | II° vector, EGM NA | SS | No AE; CXR on readmission 90° PG rotation | reprogramming to alt vector | negative FU (NDD) |
| 44-52 | 2021 [39] | 9/1254 Pt  ELISIR Registry |  | IS,  3 Pt < 48 h | NA | NA | NA | not specified, but no invasive re-intervention | NA |
| 53* | [Figure 3]  [Figure 4] | 58 M, Brugada, inducible VF,  I° prevention | 2-incision, DFT OK  CSZ 200-230 bpm | 1 IS / 24 h | I° vector: baseline drift, ↓QSR, artifacts; not reproducible | PE | AE around PE (lateral CXR) | no action (self-limiting; remote-monitoring) | negative 12 mos FU |
| 54* |  | 33 M, syncopal wide QRS tachycardia,  SCD familiarity | 2-incision, DFT OK  CSZ 200-230 bpm | 1 IS / 72 h | II° vector: baseline drift, repetitive monomorphic artifacts, post-shock EGM normalization; not reproducible | SS | NA | no action (self-limiting, delayed evaluation) | negative 12 mos FU |

AE: air entrapment; AP: anteroposterior; alt: alternate; CSZ: conditional shock zone; CXR: chest X-ray; d: day(s); DCM: dilated cardiomyopathy; DE: distal electrode; DFT: defibrillation threshold test; EGM: electrogram; F: female; FU: follow-up; GUCH: grown-adult congenital heart disease; h: hour(s); HCM: hypertrophic cardiomyopathy; HD: heart disease; IHD: ischemic heart disease; imp: impedance; IS(s): inappropriate shock(s); M: male; mos: month(s); MVT: monomorphic VT; NA: not available; NDD: non determined duration; NSVT: non-sustained VT; PE: proximal electrode; PG: pulse generator; PMVT: polymorphic VT; Pt: patient(s); SCD: sudden cardiac death; SS: set screw; TGA: transposition of the great arteries; VF: ventricular fibrillation; VT: ventricular tachycardia; wk: week(s); I° primary; II°: secondary; ↓ reduced; *: unpublished authors’ data.

**Figure S1:** **Diagnostic findings in patients with S-ICD malfunctions.**


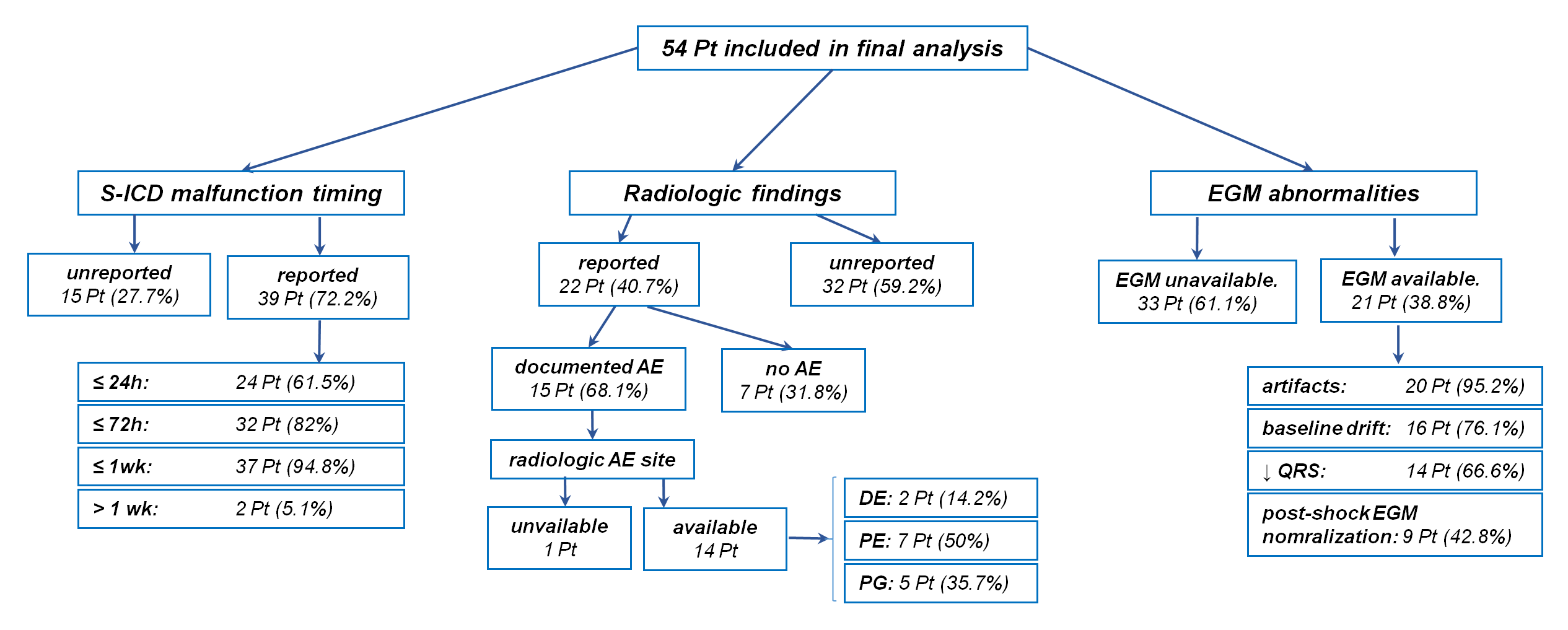


Flow-chart of available data relative to S-ICD malfunction timing, radiologic findings and EGM abnormalities in the patient population of the present review. AE: air entrapment; DE: distal electrode; EGM: electrogram; h: hour; Pt: patient(s); wk: week; PE: proximal electrode; PG: pulse generator; ↓QRS: reduced QRS amplitude.

**Figure S2:** **Patients clinical management and follow-up**.


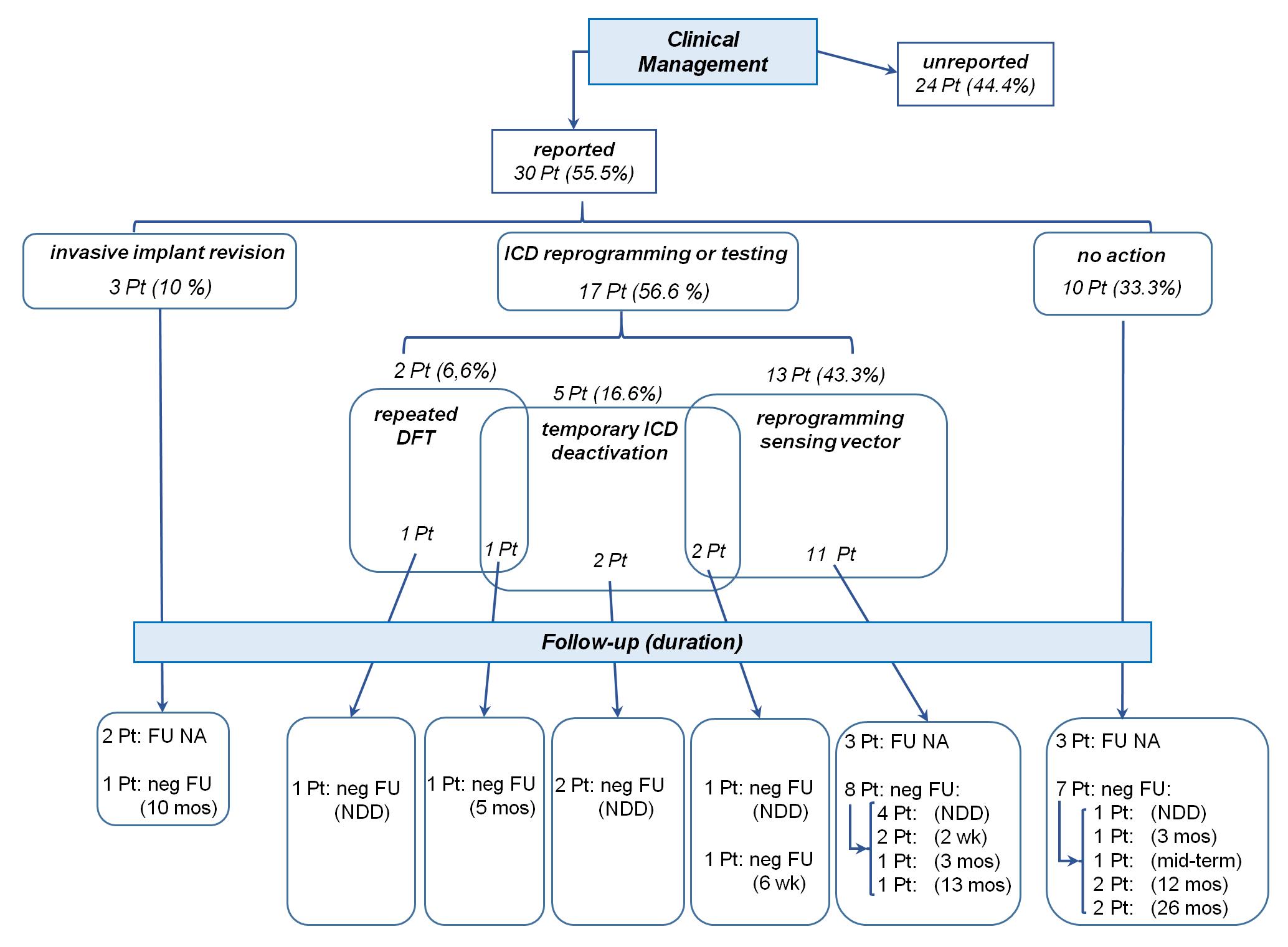


Flow-chart of available data relative to clinical management and follow-up in the patient population of the present review. DFT: defibrillation threshold test; FU: follow-up; mos: month(s); NA: not available; NDD: non-determined duration; neg: negative (no recurrence); Pt: patient(s); wk: week.
